# Regulation of pregnancy-associated plasma protein A2 (PAPPA2) in a human placental trophoblast cell line (BeWo)

**DOI:** 10.1186/1477-7827-9-48

**Published:** 2011-04-15

**Authors:** Pamela K Wagner, Aki Otomo, Julian K Christians

**Affiliations:** 1 Simon Fraser University, Biological Sciences, 8888 University Drive, Burnaby, BC, V5A 1S6, Canada

## Abstract

**Background:**

Pregnancy-associated plasma protein A2 (PAPPA2) is an insulin-like growth factor-binding protein (IGFBP) protease expressed at high levels in the placenta and upregulated in pregnancies complicated by preeclampsia and HELLP (Hemolytic anemia, Elevated Liver enzymes, and Low Platelet count) syndrome. However, it is unclear whether elevated PAPPA2 expression causes abnormal placental development, or whether upregulation compensates for placental pathology. In the present study, we investigate whether PAPPA2 expression is affected by hypoxia, oxidative stress, syncytialization factors or substances known to affect the expression of PAPPA2's paralogue, PAPPA.

**Methods:**

BeWo cells, a model of placental trophoblasts, were treated with one of the following: hypoxia (2% O2), oxidative stress (20 microM hydrogen peroxide), forskolin (10 microM and 100 microM), TGF-beta (10 and 50 ng/mL), TNF-alpha (100 ng/mL), IL-1beta (100 ng/mL) or PGE2 (1 microM). We used quantitative RT-PCR (qRT-PCR) to quantify the mRNA levels of PAPPA2, as well as those of PAPPA and ADAM12 since these proteases have similar substrates and are also highly expressed in the placenta. Where we observed significant effects on PAPPA2 mRNA levels, we tested for effects at the protein level using an in-cell Western assay.

**Results:**

Hypoxia, but not oxidative stress, caused a 47-fold increase in PAPPA2 mRNA expression, while TNF-alpha resulted in a 6-fold increase, and both of these effects were confirmed at the protein level. PGE2 resulted in a 14-fold upregulation of PAPPA2 mRNA but this was not reflected at the protein level. Forskolin, TGF-beta and IL-1beta had no significant effect on PAPPA2 mRNA expression. We observed no effects of any treatment on PAPPA or ADAM12 expression.

**Conclusion:**

Our study demonstrates that factors previously known to be highly expressed in preeclamptic placentae (PGE2 and TNF-alpha), contribute to the upregulation of PAPPA2. Hypoxia, known to occur in preeclamptic placentae, also increased PAPPA2 expression. These results are consistent with the hypothesis that PAPPA2 is upregulated as a consequence of placental pathology, rather than elevated PAPPA2 levels being a cause of preeclampsia.

## Background

Preeclampsia affects approximately 1 in 15 pregnancies [1] and is characterized by a sudden rise in blood pressure and proteinuria which resolves after delivery [2,3]. Currently, there is no effective treatment for preeclampsia other than to induce delivery. However, premature delivery elevates the risk of neonatal death and health problems later in life [3-5]. The etiology of preeclampsia is thought to involve abnormal placental development [6-8].

Numerous studies have attempted to elucidate the mechanisms underlying abnormal placental development using microarrays to identify genes differentially expressed between placentae from healthy and complicated pregnancies [9,10]. Five studies have shown that placental expression of pregnancy-associated plasma protein A2 (PAPPA2) is upregulated at delivery in preeclampsia or a complication of preeclampsia, HELLP (Hemolytic anemia, Elevated Liver enzymes, and Low Platelet count) syndrome [9,11-14]. PAPPA2 is an insulin-like growth factor-binding protein (IGFBP) protease [15] expressed at high levels during pregnancy [16,17] which shares approximately 40% amino acid similarity with PAPPA [15]. Abnormally low PAPPA levels in first trimester maternal circulation are associated with increased risk of preeclampsia [18,19], a pattern also observed for another IGFBP protease, ADAM12 [19-23].

It is not known whether altered PAPPA2 expression causes preeclampsia or is a response to placental pathology. It has been hypothesized that the upregulation of PAPPA2 in preeclamptic pregnancies is a compensatory response to abnormal placentation which might increase IGF availability and promote feto-placental growth [18]. A number of factors might trigger this upregulation, and in the present study we attempt to identify factors that regulate PAPPA2 expression.

Early placental development takes place in a low-oxygen environment [24], but impaired invasion into the decidua is thought to lead to even lower oxygen levels (hypoxia) in preeclampsia [1,10,25-27], which we hypothesize could upregulate PAPPA2. Ischemia-reperfusion injury might also occur due to intermittent blood flow into the intervillous spaces of the placenta [28], leading to the formation of damaging reactive oxygen species (ROS); this oxidative stress can be mimicked by peroxides [25,28,29].

It has been suggested that improper trophoblast cell fusion, or syncytialization, may be a cause of preeclampsia [6]. Since PAPPA2 is expressed in the syncytiotrophoblast [14,17], we hypothesize that factors that affect syncytialization [30-32] will affect PAPPA2 expression.

The paralog of PAPPA2, PAPPA, is upregulated by TNF-α, TGF-β, PGE_2_, and IL-1β in osteoblasts [33], and we predict that these factors may also regulate PAPPA2 expression, given the homology between PAPPA and PAPPA2.

The goal of the present study was to test our hypotheses regarding the regulation of PAPPA2 using BeWo cells as a model of placental trophoblasts [34,35]. In addition to PAPPA2, we also measured levels of PAPPA and ADAM12 since they are also placental IGFBP proteases [21,36] associated with preeclampsia and intrauterine growth restriction (IUGR) [19,22,23,37,38], and IGFBP proteases may be coregulated [39]. We also measured levels of E-cadherin mRNA to determine the level of syncytialization, since E-cadherin expression decreases as cells fuse [40].

## Methods

### Cell culture and materials

BeWo cells, a gift from Dr. Andrée Gruslin of the University of Ottawa, were cultured in Ham's F-12K medium (Sigma Aldrich, St. Louis, MO) supplemented with 10% fetal bovine serum, 2 mM L-glutamine, 100 U/mL penicillin and 100 U/mL streptomycin in 5% CO_2 _and 95% air at 37°C. Cells were used at passages 30-42. Porcine TGF-β, recombinant TNF-α, and recombinant IL-1β were purchased from R&D Systems (Minneapolis, MN), forskolin and PGE_2 _were purchased from Sigma (St. Louis, MO), and 3% w/v H_2_O_2 _was purchased from Anachemia (Lachine, Quebec).

### Experimental treatments

For the hypoxic treatment, cells were cultured in 9 cm plates at 30% confluence and were maintained at 37°C in 5% CO_2 _and 95% air (normoxia) for 24 h, at which time the cells had reached 50% confluence. Half of the plates (N = 5) remained under normoxic conditions while the other half were transferred to a chamber with an atmosphere of 93% N_2_, 5% CO_2_, and 2% O_2_, as used previously to simulate hypoxia during preeclampsia [31,41,42]. These plates were incubated for a further 24h before protein analysis or collection for RNA isolation.

For the growth factor and peroxide treatments, cells were cultured under normoxic conditions as described above for 24 h. The medium was then replaced with fresh medium (control samples) or medium containing H_2_O_2 _at 20 μM [29] or one of the following factors previously shown to upregulate PAPPA in osteoblasts cells: TGF-β (10 ng/mL and 50 ng/mL), TNF-α (100 ng/mL) IL-1β (100 ng/mL), prostaglandin E_2 _(1 μM), forskolin (10 μM and 100 μM, each dissolved in 0.1% ethanol) or a forskolin control (medium with 0.1% ethanol only) [30,32,33,43] (N = 5 plates per treatment).

### Cell counting

BeWo cells were cultured on 6-well plates for 24 hours, with the treatments described above. 2 μL of Hoechst 33342 (Invitrogen, Carlsbad, CA) was added (final concentration of 1.5 μg/mL), and the plates were incubated for 20 minutes at 37°C. The medium was then replaced with 2 mL of Hank's buffered salt solution (HBSS). Five pictures were taken of each well at 10x magnification, and cells were counted using ImageJ software (NIH). Each experiment was repeated three times.

### RNA isolation and RT-PCR

BeWo cells were collected and homogenized in 600 μL of buffer RLT (Qiagen, Ontario, Canada) using pipetting and Qiashredders (Qiagen, Ontario, Canada) and total RNA was extracted using the RNeasy Mini kit (Qiagen, Ontario, Canada) according to the manufacturer's instructions. RNA concentration was determined using a Nanodrop spectrophotometer (Thermo Fischer Scientific Inc. Waltham, MA), and each sample was diluted to a final concentration of 50 ng/μL. Reference samples were made by combining 50 μL from each sample. These reference samples were measured for each gene and were included in every assay to account for variation between assays. Quantitative reverse transcriptase PCR (qRT-PCR) was performed to compare levels of PAPPA2, PAPPA, ADAM12, and E-cadherin mRNA. Levels of β-actin [31] and GAPDH [44] were also measured and used to standardize the values of the other genes (see Table 1 for primer sequences). The qScript one-step SYBR green qRT-PCR kit (Quanta Biosciences Inc. Gaithersburg, MD) was used to reverse-transcribe and amplify each sample. Reverse transcription was carried out at 50°C for 30 minutes, followed by a PCR activation step of 95°C for 15 minutes, followed by amplification by 40 cycles of 1 minute at 94°C, 1 minute at either 50°C, 56°C or 60°C (see Table 1 for annealing temperatures for each gene), and 1 minute at 72°C, and a final extension of 10 minutes at 72°C. Each reaction was performed using a volume of 25 μL and contained 12.5 μL of reaction mix, 8.5 μL of RNA sample at 50 ng/μL, 1.5 μL (22.5 mM) of each forward and reverse primer, 0.5 μL of reverse transcriptase, and 0.5 μL of nuclease free water. Ct values were recorded for each gene, defined as the point at which the signal crossed a predetermined threshold. Each sample was measured in triplicate and in each assay, one negative control and one reference sample were included for each gene. For each gene, we performed a melting curve analysis, and the qRT-PCR products were run on a 1% agarose gel to ensure that only one amplicon of the expected size was present.

**Table 1:** Primer and probe sequences used in qRT-PCR for PAPPA2, PAPPA, ADAM12, E-cadherin, β-actin and GAPDH

Gene	Forward Primer	Reverse Primer	Annealing Temperature	Source
PAPPA2	ACTCACCCAAGAGGGCATACATGA	GCACTGAGCTGGCAAAGTAGATGT	50°C	This paper
PAPPA	GTCATCTTTGCCTGGAAGGGAGAA	AGGGCTGTTCAACATCAGGATGAC	56°C	This paper
ADAM12	CTGGGCCACAATTTCGGGATGAAT	ACTGCTGAACACCATGGGAAATGG	50°C	This paper
E-cadherin	AGCCTCTGGATAGAGAACGCATTG	GGGTGAATTCGGGCTTGTTGTCAT	50°C	This paper
β-actin	GCGAGAAGATGACCCAGGATC	CCAGTGGTACGGCCAGAGG	60°C	Knerr *et al. *(2005) [31]
GAPDH	CGGGAAGCTTGTGATCAATGG	GGCAGTGATGGCATGGACTG	56°C	Kudo *et al. *(2003) [30]

### In-cell Westerns

In-cell Westerns, an immunocytochemical assay to quantify proteins in fixed cells [45], were performed for treatments which had a significant effect on PAPPA2 mRNA levels (2% O_2_, 1 μM PGE_2_, 100 ng/mL TNF-α). Cells were cultured on 6-well plates, and treatments were performed as described above, using three wells per treatment (N = 3). Following treatment, the medium was removed, and the cells were fixed in 2.4 mL of 3.7% formaldehyde in PBS for 20 min. The cells were then permeabilized by washing with 3.2 mL PBS containing 0.1% Triton X-100 (Sigma Aldrich, St. Louis, MO) 5 times for 5 minutes each, with gentle shaking. The cells were blocked using 2.4 mL of near infrared blocking buffer (Rockland, Gilbertsville, PA) for 1.5 h with gentle shaking. The cells were then incubated overnight at 4°C with 1:200 goat anti-human PAPPA2 polyclonal (AF1668; R&D Systems, Minneapolis, MN) and 1:200 mouse anti-tubulin (T9026, Sigma, St Louis, MO) antibodies diluted in blocking buffer (Li-Cor Biosciences, Lincoln, NE) and 0.1% Tween-20 (Sigma, ON, Canada) with gentle shaking, followed by 5 washes with PBS and 0.1% Tween-20 for 5 minutes per wash. Incubation with the secondary antibodies was carried out using a solution containing 1:800 fluorescently-labelled IRDye 800 donkey anti-goat and IRDye 680 donkey anti-mouse secondary antibodies (Li-Cor Biosciences, Lincoln, NE) diluted in Odyssey Blocking Buffer and 0.1% Tween-20 for 1 h in the dark with gentle shaking. We performed 5 more 5 minute washes with PBS in the dark to prevent breakdown of the IR dye. All steps except for primary antibody incubations were performed at room temperature. Two negative controls were included, one omitting each primary antibody. Preliminary work showed that the PAPPA2 and tubulin signals were above negative controls under control conditions. We used tubulin to standardize the amount of cells in each well because we were unable to detect a strong signal using an anti-actin antibody (CLT9001; Cedarlane, Burlington ON). The intensity of staining in cells was quantified by densitometry using an Odyssey infrared imaging system (Li-Cor Biosciences).

### Western Blot

Cell cultured medium was centrifuged to remove cellular debris, and 40 μL was mixed with 5x SDS loading buffer and heated for 10 minutes at 100°C. Samples were run for 60 minutes through a 4% stacking and 8% separating polyacrylamide gel. After transfer, nitrocellulose membranes were blocked for 1 hour at room temperature in infrared blocking buffer, then incubated in 1:500 polyclonal goat anti-human PAPPA2 antibody diluted in the same blocking buffer, with 0.1% Tween-20. Membranes were washed 4 times in filter-sterilized PBS containing 0.1% Tween-20 at room temperature, and incubated in a solution containing 1:10000 fluorescently-labelled IRDye 800 secondary antibody diluted in blocking buffer, 0.1% Tween-20 and 0.1% SDS for 45 minutes in the dark with gentle shaking. The membranes were again washed 4 times for 5 minutes each in filter-sterilized PBS containing 0.1% Tween-20 at room temperature, rinsed with filtered PBS, and scanned with an Odyssey infrared imaging system.

### Statistical analyses

We used the method of Pfaffl [46] to calculate mRNA expression levels for PAPPA2, PAPPA, ADAM12, and E-cadherin, relative to the reference sample described above. For example, a value of 2 indicates a sample has 2 times more transcript than the reference sample, correcting for GAPDH or β-actin. qRT-PCR data were analysed using Wilcoxon signed-rank tests in JMP, Version 7 (SAS Institute Inc., Cary, NC). Results were normalized to β-actin or GAPDH.

## Results and Discussion

BeWo cells express PAPPA2, with Ct values of approximately 25 and signal above the no-primary negative control in in-cell Western analysis. We examined the mRNA levels of PAPPA2, PAPPA, ADAM12 and E-cadherin in BeWo cells treated with different factors known to be involved in preeclampsia or shown to upregulate PAPPA. GAPDH and β-actin mRNA levels were used to normalize the mRNA levels of the other genes. However, GAPDH has been shown to increase under hypoxia [47], so only β-actin was used for hypoxia. The mRNA levels of β-actin and GAPDH did not differ significantly between treatments, suggesting that they were appropriate for use as controls. When used to standardize mRNA levels, β-actin and GAPDH produced similar results.

Hoechst 33342 staining was used to assess the number of BeWo cells under each treatment. Cells with condensed staining were considered dead, whereas cells with diffuse signal were considered alive. We did not find any significant cell death between treatments, when counting either the proportion or absolute number of alive and dead cells, which is consistent with previous studies [29,31].

### Hypoxia and oxidative stress

**Figure 1:**
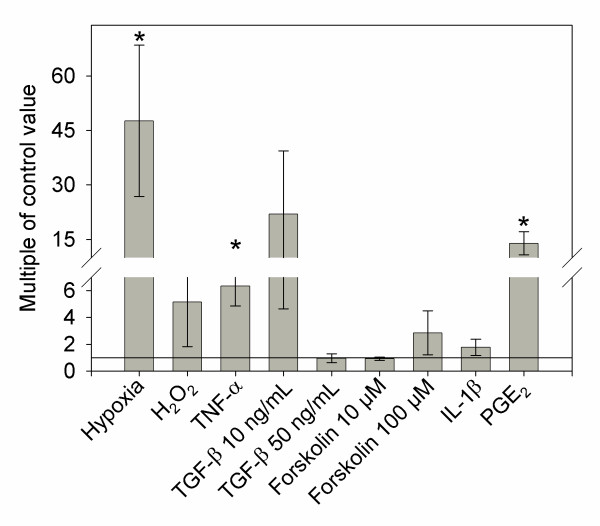
**PAPPA2 mRNA expression in BeWo cells**. PAPPA2 mRNA levels are expressed as fold change compared to control (0.1% ethanol was used as a control for forskolin). Cells were treated for 24 hours with 10 μM forskolin, 100 μM forskolin, 20 μM H_2_O_2_, 2% O_2 _(hypoxia), 100 ng/mL IL-1β, 1 μM PGE_2_, 10 ng/mL TGF-β, 50 ng/mL TGF-β, and 100 ng/mL TNF-α, N = 5. Columns with an asterisk (*) indicate expression significantly different from the control (p < 0.05). Although the mean 10 ng/mL TGF-β expression is high, only one of the five replicates showed high expression levels relative to controls.

Hypoxia has previously been shown to upregulate several factors in BeWo cells, including HIF-1 [48,49] and downregulate factors such as syncytin [44,50]. We found PAPPA2 mRNA levels were approximately 47 times higher when BeWo cells were grown under hypoxic conditions (Figure 1; Wilcoxon Z = 2.506, p = 0.0122). We confirmed that this differential expression was reflected in protein levels, and found an approximate 14% upregulation of PAPPA2 protein using in-cell Western staining. Western blotting did not detect PAPPA2 in the conditioned cell medium (data not shown), even though we were previously able to detect PAPPA2 in Western blots of murine and human tissue [17,51]. Our inability to measure secreted PAPPA2 may account for the difference in the magnitude of effect on mRNA and protein expression. Hypoxia had no significant effect on either PAPPA (Wilcoxon Z = 0.4178, p = 0.6761) or ADAM12 (Wilcoxon Z = 0, p = 1) expression. Impaired trophoblast invasion into the maternal decidua is thought to cause decreased and intermittent blood flow to the placenta [25,28,52], with reperfusion leading to an increase in reactive oxygen species with potentially damaging effects. A previous study found that addition of reactive oxygen species, specifically hydrogen peroxide at high levels (50 uM) caused an increase in apoptosis in BeWo cells [29]. We therefore used a lower H_2_O_2 _concentration (20 uM) to mimic reperfusion injury and did not find any effect on PAPPA2 mRNA expression after 24 hours (Figure 1; Wilcoxon Z = 0.417, p = 0.6761).

### Factors that affect syncytialization

TNF-α has been shown to impair syncytialization, based on impaired expression of hCG [53]. TNF-α expression has also been shown to increase in preeclamptic pregnancies at delivery [54] and in response to hypoxia/reoxygenation conditions [55]. Treating cells with 100 ng/mL TNF-α resulted in 6.34 times higher PAPPA2 expression (Figure 1; Wilcoxon Z = 2.507, p = 0.0122). This upregulation was confirmed using in-cell Western analysis, where we found an 18% upregulation in PAPPA2 protein expression compared to controls. Once again, our inability to detect free PAPPA2 in cell medium may account for the lower upregulation at the protein level. PAPPA (Z = 0.627, p = 0.5309) and ADAM12 (Z = 0.209, p = 0.8345) mRNA levels were not found to be affected by treatment with TNF-α. TGF-β has also been shown to inhibit trophoblast differentiation in human cytotrophoblast cells [56] perhaps due, in part, to its proposed tumor-suppressing activity [57]. We did not find any effect of 10 ng/mL TGF-β or 50 ng/mL TGF-β on PAPPA2 mRNA expression (Figure 1; Wilcoxon Z = 0, p = 1; Wilcoxon Z = 0.2089 p = 0.8345; respectively). Treatment with TGF-β did not cause any significant increase in PAPPA (10 ng/mL Wilcoxon Z = -0.209, p = 0.8345; 50 ng/mL Z = -0.418, p = 0.6761) or ADAM12 (10 ng/mL Wilcoxon Z = 0, p = 1; 50 ng/mL Z = 0, p = 1) mRNA levels. Furthermore, we did not find any significant difference in E-cadherin expression, suggesting that neither TNF-α (Wilcoxon Z = 0.208, p = 0.8345) nor TGF-β (10 ng/mL Wilcoxon Z = 0.626, p = 0.5309; 50 ng/mL Z = -0.418, p = 0.6761) changed the rate of syncytialization.

Forskolin has been shown to increase intracellular cAMP levels and trigger increased cellular fusion in BeWo cells [30]. Surprisingly, we did not detect a significant decrease in E-cadherin mRNA compared to the 0.1% ethanol vehicle control (10 μM: Wilcoxon Z = -1.880, p = 0.0601; 100 μM: Wilcoxon Z = -1.462, p = 0.1437), and so did not find significant evidence of syncytialization in response to forskolin treatment. However, previous studies have shown that forskolin treatment at both 10 μM [57] and 100 μM [30,32,44,58] does induce cell fusion, based on other measurements of syncytialization such as levels of syncytin and human chorionic gonadotropin (hCG). We did not find any significant effect of either 10 μM or 100 μM of forskolin on PAPPA2 expression (Figure 1; Wilcoxon Z = 0.418, p = 0.6761 in both cases).

### Factors known to upregulate PAPPA

A previous study by Conover *et al. *[33] tested the effect of various growth factors on PAPPA expression in osteoblasts, finding that TGF-β, TNF-α, IL-1β or PGE_2 _all increased PAPPA mRNA and protein expression. As we described above, in BeWo cells TGF-β had no effect on PAPPA2 expression, but TNF-α caused a significant upregulation in both mRNA and protein. Treating BeWo cells with 100 ng/mL IL-1β did not have a significant effect on PAPPA2 mRNA expression (Figure 1; Wilcoxon Z = 1.462, p = 0.1437). In preeclamptic pregnancies, PGE_2 _levels increase in placental tissue at term [59], and we found that treatment with 1 μM PGE_2 _resulted in a 14-fold upregulation of PAPPA2 mRNA levels (Figure 1; Wilcoxon Z = 2.507, p = 0.0122). However, we were not able to confirm PAPPA2 protein upregulation using the in-cell Western assay. Furthermore, PAPPA and ADAM12 were not affected by any of these factors, including PGE2 (Z = -1.6712, p = 0.0947; Z = -1.6712, p = 0.0947; respectively).

## Conclusions

Our study demonstrated that factors highly expressed in preeclamptic patients, PGE_2 _and TNF-α [54,59], increase the expression of PAPPA2, at least at the mRNA level, suggesting a potential mechanism underlying the elevated PAPPA2 levels in preeclamptic placentae at term [9,11-14]. Hypoxia, a condition known to occur in preeclamptic placentae [10], was also shown to increase PAPPA2 expression. However, these same treatments had no effect on two other IGFBP proteases, PAPPA and ADAM12. We have previously demonstrated that differences in placental PAPPA2 expression do not result in significant changes in placental or birth weight in mice [51]. Both these previous results and those of the present study are consistent with the hypothesis that PAPPA2 is upregulated as a consequence of placental pathology, rather than elevated PAPPA2 levels being a cause of abnormal placental development and preeclampsia.

## Abbreviations

ADAM12: A disintegrin and metalloproteinase 12; GAPDH: glyceraldehyde 3-phosphate dehydrogenase; HELLP: hemolytic anemia, elevated liver enzymes, and low platelet count; IGF: insulin-like growth factor; IGFBP: insulin-like growth factor-binding protein; IL-1β: interleukin-1beta; IUGR: intrauterine growth restriction; qRT-PCR: quantitative real-time polymerase chain reaction; QTL: quantitative trait locus; PAPPA: pregnancy-associated plasma protein A; PAPPA2: pregnancy-associated plasma protein A2; PGE_2_: prostaglandin E_2_; ROS: reactive oxygen species; TGF-β: Transforming Growth Factor-beta; TNF-α: tumor necrosis factor-alpha.

## Competing interests

The authors declare that they have no competing interests.

## Authors' contributions

PKW participated in the design of the study, carried out most of the experimental work and drafted the manuscript. AO performed the RNA extraction and qRT-PCR. JKC conceived of the study, participated in its design and helped to draft the manuscript. All authors read and approved the final manuscript.
